# Evaluation of a digital oral health intervention (Know Your OQ™) to enhance knowledge, attitudes and practices related to oral health

**DOI:** 10.1038/s41405-023-00166-4

**Published:** 2023-08-26

**Authors:** George Kitsaras, Juliana Gomez, Richard Hogan, Maria Ryan

**Affiliations:** 1 Dental Health Unit, Division of Dentistry, The University of Manchester, Manchester, UK; 2 Colgate-Palmolive Company, Piscataway, NJ 08854 USA

**Keywords:** Oral hygiene, Nutrition and diet in dentistry

## Abstract

**Objective/Aim:**

Optimal oral health behaviours are crucial to avoid preventable dental diseases and maintain good oral health. This research aimed to evaluate the impact of a digital oral health intervention (Know Your OQ™) in changing knowledge, attitudes and practices related to oral health.

**Materials & methods:**

Two studies were conducted with a total of 296 healthy adults. Demographic data as well as knowledge, attitudes, and practices (KAPs) related to oral health were collected before and after completion of the Know Your OQ™ intervention. The KAPs questionnaire included 19 multiple choice questions. Comprehension and feedback were also collected.

**Results:**

In total, 134 (45%) male and 162 (55%) female participants completed the two studies. Across both studies, 5 out of 7 knowledge questions and 2 out of 5 attitude questions showed significant changes pre/post-intervention with participants increasing their knowledge and improving their attitudes towards oral health. Only 1 practice changed in the first study, however, in the second study, 4 out of 7 practice questions showed significant changes pre/post-intervention. Comprehensibility was high across both studies with overall, positive feedback on the intervention.

**Conclusion:**

A digital oral health intervention was successful in increasing knowledge, changing attitudes and self-reported practices with regards to oral health in a diverse sample of the US population.

## Introduction

Oral diseases are one of the most common yet largely preventable diseases in the world [1]. In 2019, the Global Burden of Disease Study estimated that oral diseases affect around 3.5 billion people across the world, with dental caries of permanent teeth being the most common condition with an estimated prevalence of 2 billion adults and 520 million children suffering from dental caries [1]. Oral diseases, especially dental caries and periodontal disease are largely preventable through optimal, consistent and frequent oral hygiene behaviours [2]. The American Dental Association (ADA) recommends: (a) brushing teeth twice a day with a fluoride toothpaste for two minutes, (b) cleaning, e.g., flossing, between teeth daily, (c) eating a healthy diet that limits sugary beverages and snacks and (d) regular visits to a dental professional [3]. While seemingly straightforward advice, ADA’s recommendations incorporate a series of behaviours that individuals need to: (1) be aware of, (2) know when, where and how to perform these behaviours, and (3) be motivated to repeat these behaviours on a lifelong basis. It is not surprising that despite the importance of optimal oral hygiene behaviours, many people, both adults and children, struggle to follow and sustain such behaviours. In the US, it is estimated that around 50% of adults do not follow the recommended brushing frequency of twice a day [3]. While in the UK, a quarter of all adults do not meet the recommended brushing frequency [4].

Knowledge is a vital component of one’s capability to undertake and sustain a target behaviour, e.g., brushing their teeth or flossing. Capability is one of the three interconnected components, alongside opportunity and motivation, of the COM-B model of behaviour change [5]. These three factors need to be present for a given behaviour (e.g., brushing teeth, flossing etc.) to be enacted [5]. Capability is further categorised into one’s physical capability (e.g., dexterity and skills) and psychological capability, where knowledge is a vital component. Capability, either physical or psychological alone, is not enough to instigate a behaviour, however, one needs to know what to do, when, where and how to do it well [6]. To increase knowledge and in return affect psychological capability, different behaviour change techniques can be used such as: instruction on how to perform the target behaviour(s) and information about health consequences [7].

Oral health can impact multiple areas of an individual’s life, from health and wellbeing to social and overall quality of life. Oral health is linked to systemic health and mental health [8]. Raising awareness of the importance of good oral health is vital in avoiding development and progression of dental diseases with all their negative, short and long-term implications for life [9]. Despite the importance of oral health, parts of the wider population lack fundamental information, education and knowledge on oral health, for example, in the US found that less than 50% were brushing twice a day, only a quarter flossed and the frequency of their dental visits did not meet recommendations [10, 11]. Lack of awareness and limited knowledge and information on how to perform and maintain appropriate oral hygiene are detrimental in dental disease [12, 13].

Know Your OQ™ is a digital oral health intervention designed to educate and inform at the general population level, with a view to increasing knowledge of oral health. It utilises a two-pronged approach:

A. An educational website which provides information related to oral health, oral health behaviours, links between oral and systemic health, importance of maintaining optimal oral health throughout life and advice on key aspects of oral health including frequent visits to dental professionals.
B. A brief, interactive oral health quiz to test knowledge, provide feedback on each question and to provide an oral quotient (OQ) score at the end.

This intervention relies on techniques such as instruction on how to perform the target behaviour(s) and information about health consequences to increase knowledge and change attitudes around oral health; this sets the foundation for this resource to be considered as part of a wider behavioural intervention. Digital enabled interventions are gaining momentum as internet access, smartphone usage and digital literacy continue to increase among developed and developing nations [14, 15]. In the US, the UK and EU countries, on average, over 90% of adults own a smartphone, while internet access in these areas is at an all time high with over 90% of households accessing the internet [15]. The world average internet penetration is also at a historic high at 65% [16]. Digital information and education campaigns can deploy a rich learning environment for those who use them, maintaining their interest, communicating more effectively while remaining agile and adaptable.

## Aim

The aim of this research was to evaluate the effect of a digital oral health literacy intervention (Know Your OQ™, KYOQ^TM)^ in changing knowledge, attitudes and practices related to oral health. This aim was supported by the following objectives:

1. Assess the feasibility of conducting a study using a digital oral health intervention
2. Evaluate changes in knowledge, attitudes and practices related to oral health
3. Explore the comprehensibility of the Know Your OQ™ campaign and collect information on possible changes
4. Collect feedback on the intervention

## Materials and methods

### Study design

A stepped, pre-test/post-test study was conducted with a total of 296 participants. The initial evaluation of the Know Your OQ™ initiative was completed with a sample of 114 participants. A further evaluation (*N* = 182) was conducted with a more diverse (demographically and economically) sample to increase the generalisability of the results. This stepped design allowed for a greater wealth of data to be collected. Study sample sizes were in line with previous work utilising a Knowledge, Attitudes and Practices (KAPs) questionnaire in adults [17, 18] with sample sizes varying between low 100 s and high 200 s.

### Sample & recruitment

For the first study, convenience recruitment took place with participants who have expressed interest for research studies in the past approached with information about the study. In the second study, purposive sampling took place where participants, from a similar list of potential participants with expression of interest for research work, were approached in targeted recruitment in terms of gender, education level, ethnicity, age, presence of oral health problems. Purposive recruitment took into consideration the make up of general US population aiming for a more balanced representation of participants with different educational, employment and ethnic backgrounds. Inclusion criteria for both studies included: (a) ability to provided informed consent, (b) participants aged 18–65 years old (inclusive), (c) availability for the duration of the study, (d) English fluency (ability to speak, read and understand English during information provision and before consenting) and (e) access to laptop, tablet or computer with internet/data connection.

Recruitment took place across the US in the states of Arizona, Illinois, Florida, Pennsylvania and Texas. A secure research participants database was accessed and narrowed down based on the specific demographics needed for the study. Potential participants were contacted via email, text, or phone inviting them to take the online preliminary screening questionnaire (to assess inclusion criteria). Prequalified candidates were then confirmed, consented and scheduled to participate in the research. Participants were compensated for their participation in the study. Ethical approval was granted by the US IRB (USBU.S.IRB2022CP/05). Each individual provided signed, informed consent.

### Data collection methods

All participants completed a demographics form and questionnaire on knowledge, attitudes and practices (KAPs). Demographics form included questions on age, gender, employment status, ethnicity, education level and a question on previous and ongoing experience with oral health problems. KAPs included 19 multiple choice questions (7 on knowledge, 5 on attitudes and 7 on practices). Knowledge questions focused on participants’ awareness regarding some key characteristics of oral diseases, awareness on key information on oral hygiene practices and the importance of good oral health. Attitudes examined participants’ beliefs on a series of issues related to the importance of oral health and the maintenance of appropriate oral hygiene and finally, practices explored participants’ day-to-day actions with regards to their oral hygiene regime including questions on frequency of brushing, flossing, use of mouthwash and visit to dental professionals. KAPs questionnaires are frequently used within research to establish participants’ knowledge, attitudes and practices around different health practices including oral health mostly with children, adolescents or specific population groups (i.e., expecting mothers, diabetes patients etc.). The version of KAPs used in the present study is an adapted version from different KAPs questionnaires used in two previous studies both with adults [17, 18]. Supplementary Material A presents an overview of the KAPs questionnaire.

Following the intervention, participants were asked to complete a comprehension and feedback survey. These surveys assessed the comprehensibility of the intervention while also gathering information on participants’ views of strengths and areas of improvement for the intervention to inform future changes. Finally, an online feedback session was conducted with a sub-sample of participants (convenient sampling, all participants were invited on a first come, first-served basis until data saturation was achieved) in both studies to gather more information and feedback regarding their experience. Supplementary Material B provides an overview of the feedback session guide.

### Intervention

The Know Your OQ™ intervention consisted of a website with oral health information to help participants to understand and improve their oral health practices. The quiz produced a total score at the end, their “oral health quotient / OQ” designed to provide feedback on their knowledge regarding oral health. The website provides educational resources to consumers to improve oral hygiene, encourage healthier habits and promote overall systemic health. Within the website participants are able to take an interactive assessment to determine their OQ score. The Know Your OQ™ quiz consisted of 10 questions with multiple answers. The maximum score that can be achieved is 10.

### Participants’ journey

All participants were asked to complete 3 questionnaires online using Compusense software in 3 separate visits. At baseline, participants completed the demographic and pretest KAPs questionnaires. For the intervention, participants received a link to the Know Your OQ^TM^ website (https://www.knowyouroq.com/) and were asked to (1) read the information provided on the website and (2) to complete the Know Your OQ^TM^ quiz. After these steps were completed, participants received a questionnaire which explored comprehension/readability and general feedback on both the webpage and the Know Your OQ^TM^ survey. One week later, participants completed the posttest KAPs questionnaire.

### Statistical analysis

Descriptive statistics were used to summarise demographic information. McNemar’s tests were used to determine whether the pretest-posttest differences in knowledge and attitudes were statistically significant. T-tests were used for changes in practices. Descriptive statistics were used for comprehensibility and feedback data. All statistical analyses were performed using SAS (Version 9.4).

## Results

### Sample characteristics

Across the two studies, 296 participants were recruited. Table 1 presents an overview of the sample and their key characteristics. Study 2 was intentionally focused on a more demographically diverse sample to increase the representativeness of the overall study population (in comparison to the general US population). In total across both studies, 296 participants took part with the majority (*N* = 162) being females, with some college education or higher (such as postgraduate education), in full employment (*N* = 164) and from a white ethnic background (*N* = 186). Contrary to study 1, study 2 participants included higher percentages of different demographics such as a more ethnically diverse sample closer to national US demographics, for example, in study 2 15% of participants were black or African-American close to the average 13.6.% of the US population.

**Table 1:** Sample characteristics.

		Study 1 *N* = 114	Study 2 *N* = 182
Gender	Male	57 (50%)	77 (42%)
	Female	57 (50%)	105 (58%)
Education	Postgraduate education	24 (21%)	0 (0%)
	Higher education graduate or Currently in college	56 (49%)	33 (18%)
	Some college education	0 (0%)	92 (51%)
	High School graduate	34 (30%)	51 (28%)
	Some high school education	0 (0%)	6 (3%)
Employment Status	Full-time employed	80 (70%)	84 (46%)
	Part-time employed	12 (11%)	27 (15%)
	Self-employed	9 (8%)	17 (9%)
	Stay at home parent	6 (5%)	11 (6%)
	In training/education	2 (2%)	6 (3%)
	Unemployed	3 (3%)	14 (8%)
	Retired	2 (2%)	23 (13%)
Ethnicity	White	90 (90%)	96 (53%)
	Black or African-American	15 (15%)	28 (15%)
	American Indian or Alaskan Native	0 (0%)	1 (1%)
	Asian	2 (2%)	39 (21%)
	Native Hawaiian or other Pacific Islander	0 (0%)	9 (5%)
	Multiple ethnicities	3 (3%)	9 (5%)
	Other	4 (4%)	0 (0%)
Experience with oral health problems	Yes	36 (32%)	99 (54%)
	No	73 (64%)	75 (41%)
	Don’t know	5 (4%)	8 (4%)
If yes, is it an ongoing issue?	Yes	14 (39%)	59 (60%)
	No	22 (61%)	40 (40%)

### Retention and completion rates

Across both studies, there were no dropouts resulting in a 100% retention and completion rate.

### Knowledge, attitudes and practices (KAPs) changes

Study 1 (*N* = 114), saw significant improvements in some questions on knowledge and attitudes regarding oral health. With regards to knowledge, there was a 30 percentage point (ppt) increase in awareness regarding dental cavities being the most common disease in the world t(113) = 25.93, *p* = 0.000. However, still only 48% of participants were able to recognise dental cavities as the most prevalent disease post-test (increase from 18% to 48%). There was a 33ppt increase in knowledge regarding oral health affecting general health (89% of participants post-test) t(113) = 32.60, *p* = 0.000, 18ppt increase in knowledge regarding symptoms of oral disease (89% of participants post-test) t(113) = 12.90, *p* = 0.000, 12ppt increase in the knowledge regarding causes of bad breath (44% of participants post-test) t(113) = 3.89, *p* = 0.048 and 19ppt increase in awareness on the relationship between oral health and mental health (94% of participants post-test) t(113) = 20.05, *p* = 0.000. No significant changes were found on questions related to the link between oral and general health and strongest risk factors for oral cancer. However, in both cases and both studies, participants showcased a very high level of awareness at baseline, for example regarding links between oral and general health, in study 1 96% and in study 2 98% of the sample was already aware of the link between the two.

With respect to attitudes, 2 questions showed improvements pre- and post-intervention, with 17ppt improvement in attitudes on the importance of fluoride toothpaste (92% of participants ‘agreed’ or ‘strongly agreed’ post-test) t(113) = 15.43, *p* = 0.000 and a 9ppt improvement in attitudes regarding the importance of brushing twice a day (95% of participants ‘agreed’ or ‘strongly agreed’ post-test) t(113) = 5.14, *p* = 0.023. There were no significant changes in attitudes with regards to the role of sugar in dental decay, importance of frequent dental visits and the effect of plaque on dental decay. All those attitudes were already very positive with only marginal, positive changes pre/post-intervention.

Finally, in study 1, there were changes only to one practice (use of mouthwash) with a 7ppt increase in self-reported, twice-daily usage of mouthwash t(113) = 4.08, *p* = 0.43 with only a quarter of participants reporting use of mouthwash twice a day post-test. There were no further changes in practices related to oral health.

Study 2 (*N* = 182) saw similar significant changes in knowledge pretest-posttest: there was a 20ppt increase in awareness regarding dental cavities being the most common disease in the world t(181) = 19.76, *p* = 0.000. However, still only 44% of participants were able to recognise dental cavities as the most prevalent disease post-test. In total, there was a 35ppt increase in knowledge regarding oral health affecting general health (90% of participants post-test) t(181) = 48.96, *p* = 0.000, 20% increase in knowledge regarding symptoms of oral disease (90% of participants post-test) t(181) = 25.52, *p* = 0.000, 10ppt increase in the knowledge regarding causes of bad breath (44% of participants post-test) t(181) = 6.57, *p* = 0.010 and a 16ppt increase in awareness on the relationship between oral health and mental health (95% of participants post-test) t(181) = 24.74, *p* = 0.000. As with the first study, there were no significant changes found on questions related to the link between oral and general health and the strongest link to oral cancer.

For attitudes, in line with study 1, there was a 14ppt improvement in attitudes on the importance of fluoride toothpaste (80% of participants ‘agreed’ or ‘strongly agreed’ post-test) t(181) = 13.40, *p* = 0.000 and a 9ppt improvement in attitudes regarding the importance of brushing twice a day (95% of participants ‘agreed’ or ‘strongly agreed’ post-test) t(181) = 4.08, *p* = 0.043. As in the first study, there were no significant changes in attitudes with regards to the role of sugar in dental decay, importance of frequent dental visits and the effect of plaque on dental decay. All those attitudes were already very positive with only marginal, positive changes pretest-posttest.

Whilst the first study demonstrated only one significant change in practices, there were significant improvements pre/post-intervention on 4 practices related to oral health. There was a 12ppt improvement in self-reported, twice-daily brushing t(181) = 13.78, *p* = 0.000, (from 55% to 77% of participants). Also, there was a 6ppt increase in participants reporting intention to visit their dental professional once every 6 months (48% of participants post-test) t(181) = 5.88, *p* = 0.015, there was a 10% increase in self-reported use of a fluoride toothpaste when brushing post-test t(181) = 11.13, *p* = 0.000 (81% of participants posttest). Finally, there was a 6ppt increase in participants self-reporting twice daily flossing t(181) = 4.76, *p* = 0.029. Other practices showed no significant changes pre/post-intervention (Tables 2 and 3).

**Table 2:** Knowledge and Attitudes (KAPs) changes.

	Study 1 *N* = 114			Study 2 *N* = 182		
Knowledge	%Correct answer			%Correct answer		
	Pre	Post	PPT change	Pre	Post	PPT change
Dental caries most common disease in the world	18%	48%	+30A	24%	44%	+20A
Link between oral health and general health	96%	96%	+0	98%	99%	+1
Oral health impact on general health	56%	89%	+33A	55%	90%	+35A
Risk for oral cancer	72%	66%	−6	66%	61%	−5
Signs and symptoms of dental disease	71%	89%	+18A	70%	90%	+20A
Cause of bad breath	32%	44%	+12A	34%	44%	+10a
Link between oral health and mental health	75%	94%	+19A	79%	95%	+16A
**Attitudes**	**% Agreed or Strongly agreed**			**% Agreed or Strongly agreed**		
	**Pre**	**Post**	**PPT change**	**Pre**	**Post**	**PPT change**
Frequent consumption of sugar causes caries	96%	96%	+0	91%	93%	+2
Plaque causes gum disease and dental decay	96%	97%	+1	96%	98%	+2
Fluoride strengthens teeth	75%	92%	+17A	66%	80%	+14A
Necessary to brush teeth twice a day	89%	95%	+6a	91%	96%	+5a
Frequent visits to dental professionals necessary	95%	98%	+3	88%	92%	+4

Letter indicates statistical difference at the 95% CL (lower case) or at 99% CL (upper case) versus the specified product (Tukey’s HSD).

**Table 3:** Practices (KAPs) changes.

Frequency of brushing	Pre	Post	PPT change	Pre	Post	PPT change
On some days, I do not brush	4%	2%	−2	4%	4%	+0
Once a day	19%	18%	−1	23%	14%	−9
Twice a day	65%	68%	+3	65%	77%	+12A
More than twice a day	12%	12%	+0	7%	4%	−3
**Frequency of dental visits**	**Pre**	**Post**	**PPT change**	**Pre**	**Post**	**PPT change**
Never	2%	1%	−1	5%	5%	0
If there’s a problem	10%	10%	+0	23%	18%	−7
Once a year	26%	23%	−3	24%	21%	−3
Once every 6 months	61%	64%	+3	42%	48%	+6a
Once every 3 months	2%	3%	+1	5%	8%	+3
**Use of fluoride toothpaste when brushing**	**Pre**	**Post**	**PPT change**	**Pre**	**Post**	**PPT change**
	71%	78%	+7	71%	81%	+10%A
**Frequency of flossing**	**Pre**	**Post**	**PPT change**	**Pre**	**Post**	**PPT change**
Not at all	33%	26%	−7	30%	19%	11A
Once a day	55%	61%	+6	55%	60%	+5
Twice a day	9%	9%	+0	9%	15%	+6a
More than twice a day	3%	4%	+1	5%	5%	+0
**Frequency of using mouthwash**	**Pre**	**Post**	**PPT change**	**Pre**	**Post**	**PPT change**
Not at all	33%	31%	−2	34%	28%	−6
Once a day	8%	8%	+0	27%	29%	+2
Twice a day	28%	25%	+7a	18%	24%	+6
More than twice a day	2%	2%	+0	1%	1%	+0
Several times a week	30%	25%	−5	20%	17%	−3
**Rinse after brushing**	**Pre**	**Post**	**PPT change**	**Pre**	**Post**	**PPT change**
	87%	87%	+0	92%	90%	−2%
**Food/drink after brushing**	**Pre**	**Post**	**PPT change**	**Pre**	**Post**	**PPT change**
Yes, water	35%	36%	+1	35%	41%	+6
Yes, milk	0%	0%	+0	0%	1%	+1
Yes, other than water/milk	7%	4%	−3	13%	10%	−3
No	48%	54%	+6	45%	43%	−2
I do not brush my teeth at night	10%	6%	−4	8%	4%	−4

Letter indicates statistical difference at the 95% CL (lower case) or at 99% CL (upper case) versus the specified product (Tukey’s HSD).

*PPT* Percentage Point Change.

### Comprehensibility and feedback

With regards to comprehensibility, 90% and 92% of participants found the website and quiz easy to navigate and understand, respectively, across both studies. This level of feedback suggests the website and quiz offer easy, practical and intuitive design, an important component of digital interventions. As for general feedback across both studies, 58% & 59% of participants indicated they would like more practical information on how to brush their teeth and floss, respectively, to further complement the information provided. Finally, 46% of participants found the combined effect of the website and the quiz as more impactful.

## Discussion

This digital oral health intervention was successful in changing some areas of knowledge, attitudes and practices related to oral health. The intervention was positively perceived by participants leading to high retention and good engagement. As digital oral health interventions gain momentum, this study showcases some of the possible applications and impact of these interventions, particularly within larger behavioural interventions.

### Effect on KAPs

Knowledge, attitudes, and practices (KAPs) related to oral health, in most cases, significantly changed pre/post-intervention. Knowledge is an important parameter for overall oral health. According to the World Health Organisation, oral health literacy is “the degree to which individuals have the capacity to obtain, process and understand basic health information and services needed to make appropriate oral health decisions” [18]. Higher oral and general health literacy is linked to better outcomes. Knowledge regarding different conditions, how they manifest themselves, awareness of consequences, how to access and utilise services are all vital components for increased oral health literacy to prevent and treat disease [18]. Interestingly, whilst there were significant increases pretest-posttest in the numbers of participants recognising dental caries as the most common disease in the world, over half of the participants (54%) still failed to answer this question correctly posttest. Lack of awareness of the high prevalence of oral diseases can be detrimental for one’s ability to recognise the disease and its symptoms and act in a timely manner to prevent progression of disease and more complicated and costly treatment [19].

Also, the lack of significant results on questions such as the main causes of oral cancer require consideration for future iterations of this intervention. Awareness of common risk factors for oral cancer, namely tobacco and alcohol use, is important to support early recognition of symptoms and for accessing timely care. It is estimated that approximately 30% of patients wait more than three months before consulting a healthcare professional about signs of oral cancer, with the delay mainly driven by a lack of awareness and misattribution of symptoms of oral cancer [20, 21]. In this study, the data seems to suggest that the quiz’s focus on oral hygiene for many of the questions confers a form of bias on participants that ‘poor oral hygiene’ is always likely to feature in the ‘correct answer’. This can be addressed with revisions to the quiz moving forward, for example, with rewording of the possible answers.

With regards to attitudes, the two attitudes showing important changes pretest-posttest centred on the importance of fluoride toothpaste and the need to brush teeth twice a day. Lack of change across other attitudes such as the role of sugar in dental decay, importance of frequent dental visits and effect of plaque on dental decay is not necessarily a shortcoming of the current study. At baseline, these attitudes showed very high, positive scores reflecting a general, underlying, shared understanding of the importance of these areas with respect to oral health. With regards to the importance of fluoride, despite the strong evidence base and widespread advocacy for its caries-preventive effects [22, 23], there is an established, and in some geographies, growing resistance to utilise fluoride containing products, including in communities of lower socioeconomic status who may present a higher disease burden [21, 24]. It is, therefore, encouraging to observe the improvement in attitudes achieved in this study, given the public health importance of regular usage of fluoride-containing toothpastes.

When coupled with changes in practices regarding oral health, changes in knowledge and attitudes maximise their effect. Across both studies, practices represent the area of greatest focus, moving forward, to strengthen this intervention. Sustained behaviour change requires more than simple instructions on what to do [2]; considering the COM-B model, one needs to have all three components (capability, opportunity and motivation) to undertake a target behaviour. That, in part, explains the lack of significant changes in some practices. In the first study, self-reported twice-daily usage of mouthwash increased, however, still only a quarter of all participants reported using mouthwash twice a day. In the second study, 4 practices were improved including self-reported frequency of brushing with fluoride toothpaste, flossing and intention to visit a dental professional every 6 months. Nevertheless, the act of enhancing knowledge through information provision, education on topics such as health consequences, and techniques such as instruction on how to perform the target behaviour(s) can still account for some initial changes to one’s practices, as observed in this study. To achieve and sustain greater changes in practices, further enhancement of this intervention should consider the opportunity and motivation aspects of the COM-B model; this will better match the intervention to the complex drivers of oral health practices.

Between the two studies, most results were replicated with the more homogenous and the more diverse sample, for example on knowledge and attitudes towards oral health with practices improving more greatly in the second, more diversely sampled study. Stability and replication of results across both studies highlights how widespread and common misinformation and problematic attitudes on oral health as well as lack of optimal oral health practices can be across demographic divides. However, and based on the results of these studies, still in the more diverse sample, some issues were more clearly present, for example the intention to frequent dental visits where at follow-up, only 48% of study 2 participants (compared to 64% of study 1 participants) will attend a dental professional twice a year with more study 2 participants (18% compared to 10%) stating a more reactive (“when issues arise”) rather than proactive stance on dental visits. This disparity does not come as a surprise given wider issues of equitable, affordable and accessible dental care both in the US and around the world.

Finally, even though demographic characteristics did not affect scores related to knowledge, attitudes and practices, it is important to note that demographic characteristics can have important implications for one’s knowledge around oral health, attitudes towards oral health and their practices. Strong socio-demographic gradients are evidence within oral health research with those from underserved populations more exposed to lower oral health literacy and suboptimal oral health behaviour [25, 26]. Even though these differences based on socio-demographic factors manifest on individual level practices and knowledge they stem from wider societal, structural, political and economic factors beyond the immediate control of the individual [27, 28].

Oral health is integral to general health. Periodontal disease, for example, is associated with general health conditions such as cardiovascular disease and diabetes. Those with systemic disease of inflammatory nature are at greater risk of manifestation and/or progression of chronic oral diseases, such as periodontal disease, which, in turn, further complicate their overall health [28]. The goal of oral health education should be to improve knowledge, which may lead to adoption of favourable oral health attitudes and practices that contribute to healthy lifestyles and promote health.

### Strengths and limitations

This study has a series of strengths including: the large sample size, the collection of comprehensibility and feedback from participants, the use of a diverse sample reflecting the population composition of the US and the follow-up period of 7-days before the completion of KAPs to compare with baseline. With respect to limitations, a longer follow-up period would permit observation of whether changes in KAPs are sustained for a longer time period. Furthermore, the inclusion of repeated measurements across a longer follow-up period would permit exploration of fluctuations in KAPs over time. More robust evaluation of the KAPs instrument used for this study could also improve future outcomes as well as the deployment of validated instruments to quantify participants’ KAPs. Also, at pre-test, some participants’ attitudes were already scoring highly with the possibility of predisposing them to better practices. More nuanced measurements and analyses of KAPs pre- and post-test should be considered to disentangle effects in future work. Whilst the digital nature of this intervention is a success, it precludes the use of more objective measurements in areas such as oral hygiene behaviours, increasing the reliance on self-reported measures from participants. Addition of control groups in future work can better explore the causes behind changes in measurements pre and post-test. As an industry-funded study, this work could be open to conflicts of interest given the intervention examined, however, a transparent and robust process was in place to ensure an objective and accurate outcome. Finally, the intervention itself may have excluded those with limited English proficiency, no digital literacy skills, no access to smartphone/laptop and/or lack of stable internet connection.

### Future directions

Ιn its present form, this digital oral health intervention has been successful in creating initial changes in knowledge, attitudes and practices. Moving forward, improvements will be made to the content and quiz questions to reflect participant feedback, particularly in the area of specific instruction on oral hygiene behaviours such as toothbrushing and flossing. Consideration should be given to non-digital solutions for those who might lack access to digital interventions in order to be more inclusive. Whilst this initiative launched in the US, there is consideration of adaptation for other geographies for the benefit of other populations, this will require not only translations of the content, but tailoring to suit the landscape of these geographies. Finally, as highlighted earlier in this article, it is recognised that sustained behaviour change requires more than simple instructions on what to do; the evolution of this intervention should include more holistic targeting of different areas and factors affecting and impacting one’s behaviour to bolster the impact of this resource as a tool for improving oral health literacy and eliciting behaviour change. Nevertheless, this digital oral health intervention can act as a robust, agile and adaptable resource for knowledge provision and as a public health educational tool within a holistic behaviour change intervention.

## Conclusion

In this study, an oral health focused digital intervention demonstrated an ability to change knowledge, attitudes, and practices regarding oral health. Moving forward, this resource should be expanded from a behaviour change perspective and should be evaluated longitudinally in a more robust methodological design to build evidence for the impact of this initiative with respect to sustained improvement in oral health literacy and behaviours.

### Supplementary information

Supplementary material A

Supplementary material B

## Data Availability

Data will be available upon request.
