# Supplementary material for: Evaluation of a digital oral health intervention (Know Your OQ™) to enhance knowledge, attitudes and practices related to oral health
Source: BDJ Open. 2023 Aug 26;9:40. doi: 10.1038/s41405-023-00166-4 (PMC10460405; doi:10.1038/s41405-023-00166-4)
Supplement: Supplementary file 1 — Supplementary material A [file 41405_2023_166_MOESM1_ESM.pdf]

Supplementary material A

Knowledge, Attitudes and Practice (KAP) questionnaire

**Knowledge**	
What is the most common disease in the world?	Diabetes<br>Heart disease<br>Dental cavities / decay<br>Gum disease<br>Arthritis
Do you think oral health relates to general health?	Yes<br>No<br>Not sure/Don't know
How does poor oral health affect general health?	Increased risk for heart attacks<br>Increased risk for stroke<br>Increased risk for Alzheimer's disease<br>Increased risk for diabetes<br>All of the above<br>None of the above
What is the strongest risk factor for oral cancer?	Smoking/Tobacco and alcohol<br>Poor nutrition and sugar<br>Poor oral health and bad breath<br>Gender and age
What are some of the signs and symptoms of dental diseases?	Toothache<br>Swollen puffy gums<br>Blood when brushing<br>Ear pain<br>All of the above<br>None of the above
What is the most common cause of bad breath?	Asthma<br>Tonsillitis<br>Diet<br>Poor oral hygiene<br>All of the above<br>None of the above
Do you think oral health impacts your mental health?	Yes<br>No<br>Not sure/Don't know
**Attitudes**	
Do you agree or disagree that frequent consumption of sugar (candy, sweets, sugary drinks etc.) causes tooth decay?	Strongly Agree<br>Agree<br>Neither agree nor disagree<br>Disagree<br>Strongly Disagree

	Not sure/Don’t know
Do you agree or disagree that dental decay and gum disease can be caused by plaque?	Strongly Agree<br>Agree<br>Neither agree nor disagree<br>Disagree<br>Strongly Disagree<br>Not sure/Don’t know
Do you agree or disagree that fluoride toothpaste strengthens teeth?	Strongly Agree<br>Agree<br>Neither agree nor disagree<br>Disagree<br>Strongly Disagree<br>Not sure/Don’t know
Do you agree or disagree that it is necessary to brush teeth frequently?	Strongly Agree<br>Agree<br>Neither agree nor disagree<br>Disagree<br>Strongly Disagree<br>Not sure/Don’t know
Do you agree or disagree that frequent visits to dental professionals are necessary?	Strongly Agree<br>Agree<br>Neither agree nor disagree<br>Disagree<br>Strongly Disagree<br>Not sure/Don’t know
**Practises**	
How often do you brush your teeth?	Not at all<br>Once a day<br>Twice a day<br>More than twice a day
How often do you visit your dentist?	Never<br>If there’s a problem<br>Once a year<br>Once every 6 months<br>Once every 3 months
Do you use a fluoride toothpaste when brushing your teeth?	Yes<br>No<br>Not Sure/Don’t know
How often do you floss?	Not at all<br>Once a day<br>Twice a day<br>More than twice a day
How often do you use mouthwash?	Not at all<br>Once a day

	Twice a day<br>More than twice a day
Every time you finish brushing your teeth, what do you do immediately after?	Spit excess toothpaste<br>Rinse with water<br>Something else, please specify
On a normal night, after you finish brushing your teeth, do you have any food or drinks?	Yes, water<br>Yes, milk<br>Yes, food/drink other than water/milk<br>No
