# Supplementary material for: Evaluation of a digital oral health intervention (Know Your OQ™) to enhance knowledge, attitudes and practices related to oral health
Source: BDJ Open. 2023 Aug 26;9:40. doi: 10.1038/s41405-023-00166-4 (PMC10460405; doi:10.1038/s41405-023-00166-4)
Supplement: Supplementary file 2 — Supplementary material B [file 41405_2023_166_MOESM2_ESM.pdf]

Supplementary material B

Comprehension and Feedback Questions

1. Webpage

On a scale of 1-5, how easy or difficult was it to navigate and use the Know your OQ webpage?	1– Very difficult<br>2– Difficult<br>3– Neutral<br>4– Easy<br>5– Very easy
On a scale of 1-5, how easy or difficult was it to understand the information provided by the Know Your OQ webpage?	1– Very difficult<br>2– Difficult<br>3– Neutral<br>4– Easy<br>5– Very easy
On a scale of 1-5, how practical (i.e., easy to use, understand and follow) was the Know Your OQ webpage?	1– Very impractical<br>2– Impractical<br>3– Neutral<br>4– Practical<br>5– Very practical
On the webpage, there was a mention of a connected toothbrush. Do you understand what a connected toothbrush is?	Yes<br>No<br>Not sure/Don’t know
Would you like to see more instructions on how to brush your teeth on the webpage?	Yes<br>No<br>Not sure/Don’t know
Would you like to see more instructions on how to floss on the webpage?	Yes<br>No<br>Not sure/Don’t know
Is there anything missing from the webpage or something you would have liked to see?	Yes<br>No<br>Not sure/Don’t know
If yes, can you provide us with more details?	(Open text answer)

2. Quiz

Q1	On a scale of 1-5, how easy or difficult was it to complete the Know your OQ quiz?	1– Very difficult<br>2– Difficult<br>3– Neutral<br>4– Easy<br>5– Very easy

Q2	On a scale of 1-5, were the questions easy or difficult to understand?	1– Very difficult<br>2– Difficult<br>3– Neutral<br>4– Easy<br>5– Very easy
Q3	CONDITIONAL<br>Did you understand **all** the questions included in the quiz?	Yes, I understood all of the questions<br>Yes, but I struggled with some<br>No, I struggled a lot<br>Not sure/Don't know
Q3A	(If Answer is Yes, but struggled or No, I struggled a lot)<br><br>Which question(s) troubled you? If you remember the number of the question(s), then please type it in or otherwise, in a few words, describe the content of the question.	(Open text)
Q4	When you completed the quiz, do you remember the score you received?	Yes<br>No<br>Not sure/Don't know
Q5	If you remember the score, did it mean anything to you?	Yes<br>No<br>Not sure/Don't know
Q6	CONDITIONAL<br>When you completed the quiz, did you click on "Learn More" to get more information on your oral health?	Yes<br>No<br>Not sure/Don't know
Q6A	(If Answer is no)<br><br>Why was that?	Didn't see the option/Option not clear<br>No time to look at the website<br>Something else, please specify
Q7	CONDITIONAL<br>When you completed the quiz, did you click on "Sign up for updates" to get more information on your oral health?	Yes<br>No<br>Not sure/Don't know
Q7A	(If Answer is no)<br><br>Why was that?	Didn't see the option/Option not clear<br>Generally not subscribing to updates<br>Something else, please specify
Q8	Did you share the quiz or anything you learned with anyone afterward (like family, friends etc.)?	Yes<br>No<br>Not sure/Don't know
